# Mesenchymal “stem” cells, or facilitators for the development of regenerative macrophages? Pericytes at the interface of wound healing

**DOI:** 10.3389/fcell.2023.1148121

**Published:** 2023-03-02

**Authors:** Renan Fava Marson, Andrea Pereira Regner, Lindolfo da Silva Meirelles

**Affiliations:** ^1^ Graduate Program in Cellular and Molecular Biology Applied to Health—PPGBioSaúde, Lutheran University of Brazil, Canoas, Brazil; ^2^ School of Medicine, Lutheran University of Brazil, Canoas, Brazil

**Keywords:** MSCs, pericytes, perivascular cells, M2 macrophages, wound healing, mesenchymal stromal cells, mesenchymal stem cells

## Abstract

Cultured mesenchymal stromal cells are among the most used cells in clinical trials. Currently, their potential benefits include provision of mature cell types through differentiation, and secretion of various types of paracrine signaling molecules. Even though research on these cells has spanned some decades now, surprisingly, their therapeutic potential has not been fully translated into clinical practice yet, which calls for further understanding of their intrinsic nature and modes of action. In this review, after discussing pieces of evidence that suggest that some perivascular cells may exhibit mesenchymal stem cell characteristics *in vivo*, we examine the possibility that subpopulations of perivascular and/or adventitial cells activated after tissue injury behave as MSCs and contribute to the resolution of tissue injury by providing cues for the development of regenerative macrophages at injured sites. Under this perspective, an important contribution of cultured MSCs (or their acellular products, such as extracellular vesicles) used in cell therapies would be to instigate the development of M2-like macrophages that support the tissue repair process.

## 1 Introduction

In the 1970s, Dr. Alexander Friedenstein and colleagues described bone marrow cells that could give rise to fibroblastic cells when cultured (Friedenstein et al., 1970). The cell that, once removed from the bone marrow, could form a fibroblastic colony when placed in culture was called a colony-forming unit-fibroblast (CFU-F) (Friedenstein et al., 1974b), analogous to the colony-forming unit-spleen (CFU-S) described in the field of hematopoietic stem cell research (Till and McCulloch, 1961). The cultured fibroblastic cells initially described by Friedenstein and colleagues were later shown to be adipogenic (Lanotte et al., 1981), and osteochondrogenic at the single-colony level (Friedenstein et al., 1987).

In the late 1970s, researchers trying to make hematopoiesis work *in vitro* demonstrated that doing so was possible as long as these fibroblastic cells were added to the culture system to provide hematopoietic stem cells with physiological support, as the stroma in bone marrow would (Dexter et al., 1977); consequently, these cells became known as stromal cells. In the late 1980s, Dr. Maureen Owen and colleagues found data that indicated that CFU-Fs from bone marrow are a heterogeneous population containing cells with various degrees of primitiveness, and proposed that CFU-Fs comprised marrow stromal stem cells and progenitor cells that could differentiate along fibroblastic, reticular, adipocytic, and osteogenic pathways. (Owen et al., 1987; Owen, 1988). In 1991, Dr. Arnold Caplan discussed evidence for the existence of mesenchymal stem cells in the embryo, and extended that concept, on an operational basis, to culture-expanded adherent bone marrow cells able to form bone and cartilage in vivo assays (Caplan, 1991). In the years that followed, culture-expanded adherent cells obtained from the bone marrow were designated in various ways, such as marrow stromal cells, mesenchymal stem cells, mesenchymal stromal cells, skeletal stem cells, or stromal stem cells.

Up to the mid-late 1990s, the main criteria to define mesenchymal stromal/stem cells were their ability to adhere to tissue culture-treated plastic, proliferate in culture, and differentiate into osteoblasts, chondrocytes, and adipocytes; surface immunophenotyping also became a useful tool to characterize these cells (Pittenger et al., 1999). Later on, cells with these characteristics were described in various other organs/tissues, and, consequently, these cells were suggested to exist in all vascularized organs/tissues (da Silva Meirelles et al., 2006). In 2005, the International Society for Cell Therapy (ISCT) proposed that the term mesenchymal stem cell should be reserved for cells proven to fulfill strict criteria, while cells defined *in vitro* on the basis of expression of a defined set of marker molecules should be called “multipotent mesenchymal stromal cells” (Horwitz et al., 2005). This position is maintained in the most recent ISCT’s guidelines for mesenchymal stromal cell nomenclature (Viswanathan et al., 2019). To avoid confusion, in this review, we will refer to these mesenchymal cells as “mesenchymal stromal cells” (MSCs), and mesenchymal stem cells in the strict sense of this concept will be spelled out.

In the article that put forth the concept of the existence of mesenchymal stem cells in postnatal bone marrow, Dr. Arnold Caplan foresaw the use of MSCs to generate specific tissues such as cartilage or bone (Caplan, 1991). Consequently, the use of MSCs for the formation of bone (Goshima et al., 1991), cartilage (Grande et al., 1995), muscle cells (Saito et al., 1995; Fukuda, 2001), and tendon (Awad et al., 1999) was described. It seemed, then, that the most important characteristic of MSCs was the ability to provide new cells by means of differentiation. However, this view declined in the mid 2000s, when some studies showed that the main therapeutic effects of MSCs are produced by the secretion of soluble molecules that affect surrounding cells (Kinnaird et al., 2004a; 2004b; Gnecchi et al., 2005; Tang et al., 2005). At nearly the same time, the effects of MSCs on immune (mainly adaptive) cells were described (Bartholomew et al., 2002; Di Nicola et al., 2002; Krampera et al., 2003; Meisel et al., 2004) and a work suggesting their ability to suppress graft-versus-host disease came out (Le Blanc et al., 2004), which drew the attention of scientists and the public in general. In 2009, MSCs were found to produce extracellular vesicles that transfer therapeutic molecules, particularly RNA, to neighboring during tissue injury (Bruno et al., 2009). In sum, from the early 1990s to the late 2000s, the consensus on the mechanisms underlying the clinical potential significantly changed: currently, MSCs are not viewed merely as building blocks for tissue engineering, but mainly as cells able to secrete a range of signaling molecules that can contribute to wound healing.

A search at www.clinicaltrials.gov on 3 Feb 2023 using the string ["MSC” OR “mesenchymal stromal cells” OR “mesenchymal stem cells"] returned 13,004 clinical studies involving these cells, of which 4,095 were active (i.e., recruiting, enrolling by invitation or active, not recruiting). These numbers attest the therapeutic potential of mesenchymal stem/stromal cells. Paradoxically, as pointed out recently, MSCs are not widely used in the clinic yet, possibly owing to the types of disease for which MSCs have been used, and to poor knowledge on the pathology of these diseases (Mastrolia et al., 2019). A third factor that could account to this slow transition of the use of MSCs as routine therapeutic agents might be a yet not comprehensive understanding of MSC biology in the context of the organism, including the behavior of cells that give rise to MSCs, and the interaction of these cells with others for the resolution of tissue injury. These points will be approached in the next sections.

## 2 Where do the cells that give rise to MSCs come from?

In 1999, Drs. Paolo Bianco and Giulio Cossu drew attention to the fact that the predominant stromal cell type in bone marrow, the adventitial reticular cell, can give rise to adipocytes and bone *in situ* (Bianco and Cossu, 1999). Bianco and Cossu further pointed out that the adventitial reticular cell is a special type of pericyte, and that pericytes from bovine retinas and large blood vessels had been shown to be osteogenic and chondrogenic (Bianco and Cossu, 1999). The pericyte is a type of perivascular cell that, unlike other perivascular cells such as smooth muscle cells, is in direct contact with endothelial cells in blood vessels by means of peg-and-socket contacts that contain tight and gap junctions (Gerhardt and Betsholtz, 2003).

As early as 1982, pericytes had already been suggested to give rise to mature cell types *in situ*, particularly during wound healing. Richardson et al. (Richardson et al., 1982) observed sections of thermally injured adipose tissue over time, and suggested that pericytes and fibroblasts give rise to new adipocytes after the injury. Diáz-Flores and coworkers labeled vascular and perivascular cells with monastral blue and found mature osteocytes and chondroblasts bearing inclusions of this dye after experimentally inducing injury in bone and cartilage (Diaz-Flores et al., 1991; 1992). Brighton and Hunt (Brighton and Hunt, 1997) used an experimental model of bone injury to observe the behavior of pericytes, and provided morphological evidence that pericytes contribute to bone formation during bone wound healing. Dore-Duffy et al. (2000) obtained data that suggested that brain pericytes detach from blood vessel walls and migrate into the parenchyma after traumatic brain injury. Davidoff et al. (2004) found evidence that nestin-positive mural cells (vascular smooth muscle cells and pericytes) can give rise to Leydig cells in the rat testis. In parallel with these findings, others found that pericytes isolated through classical methods (isolation and culture of capillary blood vessels) behaved as MSCs in culture as judged from their ability to proliferate and differentiate along the osteogenic, chondrogenic and adipogenic lineages (Brighton et al., 1992; Doherty et al., 1998; Farrington-Rock et al., 2004). In view of these findings, various research groups adopted the working hypothesis that pericytes, or at least pericyte subpopulations, could represent mesenchymal stem or progenitor cells *in vivo*, and give rise to MSC cultures (Bianco et al., 2001; Caplan, 2008; da Silva Meirelles et al., 2008; Dore-Duffy, 2008).

## 3 Are pericytes mesenchymal stem cells?

With the suggestion that pericytes could represent mesenchymal stem cells, in the mid-late 2000s, results of further research on this subject became available. For example, in 2007, Dellavalle et al. (2007) found that, in skeletal muscle, alkaline phosphatase (ALP)-positive pericytes were myogenic progenitors distinct from satellite cells, which were positive for CD56; when cultured, ALP^+^ cells expressed pericyte markers such as the nerve/glial antigen 2 (NG2) and CD140b. In 2007, Dr. Paolo Bianco’s group demonstrated that bone marrow cells isolated from mice based on expression of CD146 and lack of expression of CD45 contained all assayable CFU-Fs, and traced the expression of CD146 to adventitial reticular cells (Sacchetti et al., 2007). Sacchetti et al. (2007) further demonstrated that these cells are able to transfer the hematopoietic niche *in vivo*, adding to the previous works of Tavassoli and Crosby (1968) and Friedenstein et al. (1974a). However, even though this work demonstrated the importance of adventitial reticular cells, it did not prove that they are stem cells *in vivo* because the numbers of the implanted cells were not accessed throughout the life of the animals that received the cell infusions. In 2008, Dr. Bruno Peault’s group isolated perivascular cells from various tissues based on the expression of CD146 and lack of expression of CD34 (an endothelial cell marker), CD45 (a hematopoietic cell marker), and CD56 (a muscle progenitor cell marker), and demonstrated that these cells could behave as MSCs when cultured, as shown by their ability to give rise to osteoblasts, chondrocytes and adipocytes under appropriate inductive conditions (Crisan et al., 2008). While these two latter works represented an advancement over the isolation of pericytes through classical methods, again, they still did not solve the problem of defining whether pericytes are stem cells *in vivo*, even though it was evident that pericytes can give rise to MSCs when removed from the body and expanded in culture.

## 4 Are pericytes mesenchymal stem cells in the body?

The development of genetic lineage tracing systems fueled the emergence of studies designed to check if pericytes could behave as mesenchymal stem cells in the body. In 2008, Tang et al. tracked the fate of peroxisome proliferator activator receptor-gamma (PPAR-γ)^+^ and CD140b^+^ mural cells; the resulting data suggested that perivascular cells give rise to adipocytes in white adipose tissue (Tang et al., 2008). Later, another lineage tracing study tracked the progeny of FoxD1^+^ pericytes in the kidney; the results indicated that FoxD1^+^ pericytes give rise to myofibroblasts and contribute to fibrosis after renal injury (Humphreys et al., 2010). In 2010, genetic labeling allowed the detection of the contribution of Osx^+^ pericytes to bone during development and after bone injury in mice (Maes et al., 2010). Also in 2010, tracking of nestin^+^ perivascular cell in the bone marrow allowed the observation of the contribution of these cells to the formation of bone and cartilage *in situ* after 8 months, but not after a short one-month chase time (Méndez-Ferrer et al., 2010). In 2011, Feng et al. studied the fate of the progeny of NG2^+^ cells in the incisive teeth of mice; they found that a subpopulation of the NG2^+^ genetically labeled cells can give rise to odontoblasts during dental wound healing (Feng et al., 2011). On that same year, Dellavalle et al. published the results of experiments that showed that ALP^+^ pericytes can give rise to skeletal muscle fibers during a short time frame after birth (Dellavalle et al., 2011). Three years later, genetically labeled perivascular cells positive for the leptin receptor (LEPR) were shown to produce adipocytes and bone cells *in situ* during development in bone marrow; these LEPR^+^ cells were also able to give rise to bone cells during wound healing after bone fracture (Zhou et al., 2014). One year later, Kramann et al. demonstrated that the genetically labeled progeny of Gli-1^+^ perivascular cells become fibrotic cells in injured organs such as the kidneys, liver, heart, and lungs (Kramann et al., 2015). Clearly, lineage tracing studies cannot be carried out in humans, but it is possible to investigate whether specific transcripts detected in perivascular cells with stem or progenitor cell characteristics described in lineage tracing studies are present in human pericytes. In this context, our group has studied the global gene expression of highly purified, non-cultured human adipose tissue-derived pericytes (da Silva Meirelles et al., 2016). The pericytes analyzed in that study had been isolated by pre-selecting cells capable to adhere to plastic for an hour in culture, and then subjecting the pre-selected cells to fluorescence-activated cell sorting to isolate those positive for the antigen defined by the 3G5 antibody while negative for the endothelial cell marker CD31 (da Silva Meirelles et al., 2015). The results indicated that these non-cultured human pericytes share the expression of genes that encode markers associated with perivascular cells shown to behave as stem or progenitor cells in lineage tracing experiments, such as LEPR, GLI-1, nestin, ZNF423, BHLHE22, CYP7B1, PTH1R, IL7, and CXCL12 (da Silva Meirelles et al., 2016).

It is important to highlight that there is also one study indicating that perivascular cells do not behave as stem or progenitor cells nor contribute to fibrosis. In 2017, after tracing the fate of the progeny of Tbx18^+^ perivascular cells, Guimarães-Camboa et al. concluded that pericytes are not mesenchymal stem cells *in situ*, neither do they contribute to fibrosis (Guimarães-Camboa et al., 2017), even though the prospectively isolated pericytes behaved as MSCs in culture, in agreement with other works (Crisan et al., 2008; da Silva Meirelles et al., 2015; Kramann et al., 2015; Supakul et al., 2019). Guimarães-Camboa et al. also found that the expression of the green fluorescent protein (GFP) label used to detect and isolate pericytes was not observable when Tbx18^+^ perivascular cells were isolated and allowed to proliferate *in vitro*, so that anti-GFP antibodies were required to demonstrate its presence in the cultured cells (Guimarães-Camboa et al., 2017). If the same happened to the genetically labeled proliferative Tbx18^+^ cells observed *in situ*, it is possible that the reporter signal was not detectable in proliferative cells derived from pericytes *in vivo* as well. Unfortunately, in that study, the authors did not use antibodies to try to detect the progeny of Tbx18^+^ cells *in situ*, so the possibility of transgene silencing in the progeny of the genetically labeled cells cannot be discarded. Additionally, in that study, the fate of pericytes that do not express Tbx18 was not evaluated, leaving room for the possibility that multipotent pericytes may exist in the Tbx18^−^ perivascular cell population. Perhaps not surprisingly, two years later, another lineage tracing study brought further evidence that the progeny of NG2^+^ pericytes do give rise to mature bone cells after bone healing (Supakul et al., 2019). And, more recently, to make matters even more complicated, Julien et al. found data indicating that paired related homeobox 1 (Prx-1)-positive cells from skeletal muscle adjacent to bone, which co-express the pericyte markers NG2 and PDGFRβ, contribute to the formation of a fibrous callus tissue that precedes bone formation in a model of bone injury (Julien et al., 2021). These data raise the question as to whether pericytes from muscle surrounding bone, rather than perivascular cells already present around bone capillaries, provide mature bone cells when an osseous lesion occurs. Clearly, further studies are warranted to provide a clear answer to this question.

An obvious limitation of the lineage tracing experiments mentioned above is that they rely on the expression of specific markers to detect the progeny of possible stem cell populations. As explained earlier in this review, the history of research in the MSC field has pointed toward perivascular cells/pericytes as possible MSCs *in vivo*. Consequently, researchers concentrated their efforts in performing lineage tracing experiments using markers known to be expressed by perivascular cells, and most genetic labeling studies seeking for mesenchymal stem cells have focused on identifying the progeny of pericytes *in vivo*. Clearly, this does not mean that no other cell types could behave as stem cells in mesenchymal tissues; for example, other cell populations, in addition to pericytes, have been found to produce differentiated progeny in mouse incisors in lineage tracing experiments (Feng et al., 2011; Kaukua et al., 2014). Additionally, the fact that specific pericyte populations have been shown to give rise to differentiated progeny *in vivo* does not mean that pericytes in different tissues are identical, as evidence indicates that their differentiation programs are tissue-specific (Pierantozzi et al., 2015; Sacchetti et al., 2016; Yianni and Sharpe, 2018).

## 5 Pericytes give rise to MSCs, but evidence that pericytes are mesenchymal stem cells is lacking

So, in view of the current evidence, is it sensible to consider pericytes as mesenchymal stem cells in the body? Before moving on to answer that question, it is important to note that pericytes are not a homogeneous cell population. Pericytes from different tissues are expected to be different to at least some extent because of their different environments. Sacchetti et al. (2016) isolated populations of perivascular cells from various tissues and found that they have different gene expression profiles and differentiation abilities according to their tissue of origin. Additionally, even in the same tissue, differences between pericytes exist. For example, in the skeletal muscle of mice, NG2^+^ and nestin^+^ pericytes behave differently: Even though both pericyte types express CD140b and CD146, only the former gives rise to adipocytes, while the latter may contribute to muscle regeneration (Birbrair et al., 2013). Grant et al. (2019) described differences between murine brain pericytes according to their position in the vascular tree, morphology, and alpha-smooth muscle actin (α-SMA) expression. These authors categorized pericytes into ensheathing pericytes (in pre-capillary arterioles), which are α-SMA^+^, and capillary pericytes, which are α-SMA^─^. Grant et al. (2019) further divided capillary pericytes into mesh pericytes and thin-strand pericytes according to their morphology. Among mural cells in human bone marrow, CD146 identifies α-SMA^+^ pericytes and vascular smooth muscle cells, whereas sinusoids contain perivascular CD271^+^, ALP^+^ cells (Flores-Figueroa et al., 2012). Another example of pericyte diversity within a single tissue comes from human adipose tissue, where both CD146^+^ (Crisan et al., 2008) and CD146^−^ (da Silva Meirelles et al., 2015) pericytes have been described, with the latter expressing message for CD146 in spite of being surface-negative for this marker (da Silva Meirelles et al., 2015). Finally, pericytes have been shown to have heterogeneous embryonic origins even in the same tissue (Dias Moura Prazeres et al., 2017).

Therefore, given the diversity of pericytes, it is unlikely that just any pericyte is a mesenchymal stem or progenitor cell in the body. On the other hand, it might be possible that some specific pericyte populations remain in the body for a lifetime while occasionally giving rise to cells at a more advanced differentiation state, a phenomenon that could occur at a higher frequency during wound healing. As seen above, in the scientific literature, there are data that suggest some pericyte populations are stem or progenitor cells *in vivo* as well as data that indicate that pericytes are not stem cells at all. The number of published works that favor the hypothesis that particular pericyte populations give rise to differentiated progeny *in vivo* is far greater than the number of works that suggest the opposite, and it is possibly too early to completely discard the hypothesis that some pericyte types may behave as stem or progenitor cells in some tissues. Most of studies that traced the fate of cells exhibiting pericyte markers mentioned above could find differentiated cells expressing the marker used, i.e., cells that descend from a given population of pericytes. However, those studies were not designed to precisely determine the numbers of those pericytes throughout the experimental period. If those specific pericyte populations are stem cells, their numbers are supposed to remain similar over time–but what if their numbers decrease?

**FIGURE 1:**
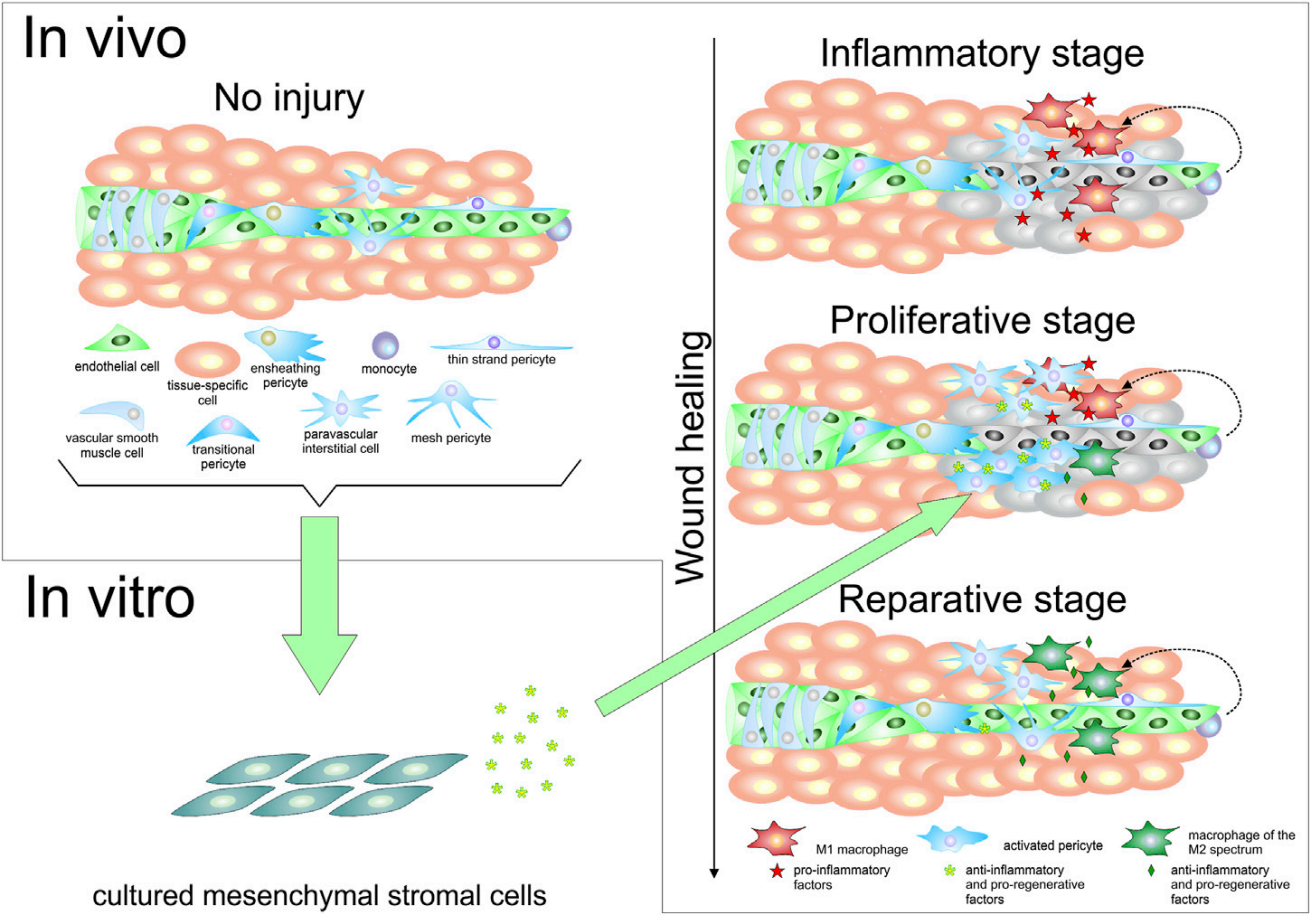
Pericytes and mesenchymal stromal cells *in vivo* and *in vitro*. The upper left portion of the figure shows a schematic representation of a portion of a tissue, where a small blood vessel formed by endothelial cells is surrounded by vascular smooth muscle cells at the arteriole level, and by different types of pericytes, including a transitional form that represents an intermediate between vascular smooth muscle cells and capillary pericytes. A paravascular interstitial cell is also represented, as well as a monocyte, and tissue-specific cells. The lower left portion of this figure represents the fact that not only pericytes, but various cell types including some mature tissue-specific cells, endothelial cells, and even monocytes have been found to be able to give rise to cells bearing mesenchymal stromal cell characteristics in culture. The right half of the figure schematically depicts the conversion of monocytes into M1 macrophages after a tissue injury event, in the inflammatory stage of wound healing (for sake of simplification, tissue-resident macrophages are not depicted). These M1 macrophages secrete pro-inflammatory factors, which lead to pericyte activation and, possibly, paravascular interstitial cell proliferation. In the proliferative stage of wound healing, the progeny of perivascular cells secrete molecules that contribute to change the balance between pro-inflammatory and pro-regenerative macrophages toward an increase of the latter type, referred to as “macrophages of the M2 spectrum”. As these reparative macrophages become more prevalent, they produce increased numbers of anti-inflammatory and pro-regenerative molecules. In the reparative stage of the wound healing process, reparative macrophages contribute to angiogenesis, which restores blood supply to the tissue; additionally, some of the previously activated pericytes may re-acquire a resting pericyte phenotype to provide physical and functional support to the newly formed blood vessels. Soluble molecules produced by cultured mesenchymal stromal cells (whether in extracellular vesicles or not), or cultured mesenchymal stromal cells themselves, can be used to favor the acquisition of a reparative phenotype by monocytes and M1 macrophages, as represented by the arrow that connects the lower left and right portions of the figure.

Ganguly et al. have demonstrated that a reduction in the number of CFU-Fs from human bone marrow along aging is associated with a decrease in the number of CD45^─/low^CD271^+^ cells (Ganguly et al., 2019), which correspond to bone marrow pericytes, aka adventitial reticular cells (Cattoretti et al., 1993). In spite of the reduction found in the number of CFU-Fs, Ganguly et al. found no evidence for decreased quality of the CD45^─/low^CD271^+^ cells in elder individuals (Ganguly et al., 2019). Inoue et al., in turn, found that cultured MSCs isolated from the adipose tissue of patients with type 2 diabetes with cardiovascular diseases had impaired proliferative capacity and inferior ability to promote angiogenesis *in vivo* compared to their counterparts from control subjects (Inoue et al., 2019). When cells from the non-cultured stromal/vascular fraction of these diabetic patients were analyzed, they were found to contain a significantly lower number of CD271^+^CD34^+^CD31^─^ cells as compared to controls (Inoue et al., 2019). In samples from human skeletal muscles, Hejbøl et al. found a population of cells co-expressing CD271, CD34, CD10, and platelet-derived growth factor receptor α (PDGFRα) “closely related to the periphery of small vessels”, which becomes amplified after muscle injury but are negative for the pericyte marker CD146 (Hejbøl et al., 2019). This phenotype is consistent with that of 3G5^+^ human adipose tissue pericytes, which were found to be positive for CD271, CD34, and PDGFRα, among other markers, but do not express CD146 on their surface (da Silva Meirelles et al., 2015; 2016). In their account on CD271^+^CD34^+^CD10^+^PDGFRα^+^ cells in human skeletal muscle, Hejbøl et al. additionally indicated that, while some of these cells were in close contact with blood vessels, others were distributed along the interstitial space (Hejbøl et al., 2019). This distribution is similar to that described for CD271^+^ bone marrow adventitial reticular cells (Cattoretti et al., 1993). In view of these findings, it is possible that some populations of CD271^+^ cells behave as mesenchymal progenitor cells in tissues such as bone marrow, adipose, and muscle, where they may exist not only as blood vessel-associated cells, but also in a paravascular/interstitial location according to particularities of these tissues (Figure 1). Currently, it is apparent that a common phenotype for this cell population in humans includes expression of CD10, CD34 (possibly absent in bone marrow CD271^+^ cells), PDGFRα, PDGFRβ, CXCL12, ALP (possibly absent in muscle CD271^+^ cells), nestin, and CD295 (leptin receptor), in addition to CD271 (Méndez-Ferrer et al., 2010; Churchman et al., 2012; da Silva Meirelles et al., 2015, 2016 Ganguly et al., 2019; Hejbøl et al., 2019; Julien et al., 2021). Evidently, further research on this subject, with the inclusion of additional tissues in the analyses, are warranted to define whether cells that bearing this phenotype could fit a mesenchymal stem cell definition, especially from the self-renewal standpoint. That said, the evidence currently available does not provide a reliable basis for the assumption that there are mesenchymal stem cells in the adult body, or that pericytes are stem cells. On the other hand, the current evidence strongly suggests that pericytes may become activated after tissue injury and give rise to MSCs that contribute to the tissue repair process, as will be further discussed below.

## 6 The crosstalk between pericytes, MSCs and macrophages during tissue injury and repair

As mentioned earlier in this review, in the mid-2000s, it became apparent that the main therapeutic effects of MSCs are produced by the secretion of soluble molecules that affect surrounding cells, rather than by provision of differentiated cells. Such a view was incorporated in a model proposed in 2008, in which pericytes become activated after tissue injury and, in this activated state, secrete a number of molecules that exert trophic and immunomodulatory effects that contribute to tissue repair (da Silva Meirelles et al., 2008). The paradigm change on the mode of action of MSCs even led to the proposition of an additional designation for these cells under the MSC acronym: medicinal signaling cells (Caplan, 2010). However, a piece of information that is sometimes overlooked when considering the therapeutic properties of MSCs is the fact that inflammatory cells also play a fundamental role in determining the course of the wound healing process (Eming et al., 2017).

One of the main cell types involved in the inflammation that follows tissue damage is the macrophage, which is represented by tissue-resident macrophages, and macrophages derived from monocytes. When activated, macrophages may take up phenotypes initially divided into two categories, namely, M1 (classically activated, pro-inflammatory) and M2 (alternatively activated, pro-regenerative) (Mantovani et al., 2002). Defining alternatively activated macrophages collectively as “M2” is clearly an oversimplification, as the M2 spectrum as defined *in vitro* comprises M2a, M2b, M2c and M2d macrophages (Table 1), which have different characteristics and acquire these phenotypes by exposure to different stimuli (Martinez et al., 2008; Ferrante and Leibovich, 2012); in view of that, one can expect the diversity of M2 macrophages *in vivo* to be even greater, given the complexity of the stimuli present.

**TABLE 1:** Macrophage phenotypes described in cultured cells.

	Pro-inflammatory macrophages	Pro-regenerative macrophages			
Phenotype	M1	M2a	M2b	M2c	M2d
Inducers	IFN-γ, LPS	IL-4, IL-13	ICs, IL-1β, LPS	IL-10, TGF-β, GCs	adenosine
Molecules secreted	IL-1β, IL-6, IL-12, TNF	IL-10, IGF-1, EGF, TGF-β	IL-10, TNF, IL-1β, IL-6	IL-10, TGF-β, EGF	IL-10, VEGF

This table was compiled with information from Martinez et al. (2008), Ferrante and Leibovich (2012), and Hesketh et al. (2017). IFN-γ, interferon gamma; LPS, lipopolysaccharide; IL, interleukin; ICs, immune complexes; TGF-β, transforming growth factor beta; GCs, glucocorticoids; TNF, tumor necrosis factor; IGF-1, insulin-like growth factor 1; EGF, epidermal growth factor; VEGF, vascular endothelial growth factor.

Inflammation during wound healing usually occurs in three different steps: an initial proinflammatory stage, followed by a reparative stage, and a final stage when inflammatory cells undergo apoptosis or leave the injured site (Eming et al., 2017). In this context, macrophages of the M2 spectrum that arise during the tissue repair process are fundamental to suppress inflammation, stimulate matrix production, and promote angiogenesis by means of production of molecules such as interleukin 10, transforming growth factor β (TGF-β), and vascular endothelial growth factor, respectively (Hesketh et al., 2017).

Whereas cultured pericytes, which in essence represent MSCs, have been shown to exert effects that are generally immunosuppressive on adaptive immune cells (Navarro et al., 2016), resting pericytes in their native microanatomical locations have been shown to interact with innate immune cells in various ways, such as facilitating the movement of neutrophils through and around the endothelium of blood vessels (Proebstl et al., 2012), and even attracting and stimulating these cells after exposure to lipopolysaccharide (LPS) (Stark et al., 2013). In the lungs of mice, pericytes have been shown to increase production of pro-inflammatory molecules six hours after LPS-induced inflammation (Hung et al., 2017). Therefore, pericytes stimulate inflammation at the beginning of injury. However, this pro-inflammatory state soon changes to an anti-inflammatory state. Minutti et al. (Minutti et al., 2019) demonstrated, in mice, that the inflammation that takes place after tissue injury is associated with secretion of amphiregulin by tissue-resident macrophages, which makes pericytes produce the active form of transforming growth factor-β (TGF-β). The biologically active form of TGF-β not only suppresses type 1 inflammation (Eming et al., 2017), but also leads to pericyte activation (with consequent proliferation) and differentiation into myofibroblasts, which secrete extracellular matrix proteins that contribute to wound healing (Minutti et al., 2019). In this context, activated pericytes have a behavior similar to that of cultured MSCs, which have been shown to be immunosuppressive owing to secretion of TGF-β back in 2002 (Di Nicola et al., 2002). Additionally, MSCs were found to promote a switch from an M1 to an M2 polarization in macrophages when administered to experimental models of injury (Li Y. et al., 2017a; Li et al., 2017b; Yin et al., 2018). Macrophage polarization toward an M2 phenotype can also be achieved by using culture medium conditioned by MSCs (Guillén et al., 2018) or MSC-derived extracellular vesicles (Lo Sicco et al., 2017). In considering the thesis that cultured MSCs are similar to activated pericytes, it is possible to theorize that an important part of the contribution of pericytes to the establishment of a regenerative niche after tissue injury occurs through the modulation of the phenotype of macrophages as a result of a dynamic crosstalk between these two cell types, as depicted in Figure 1. Under this perspective, one of the main roles of activated pericytes during wound healing would be contributing to the development of macrophages of the M2 spectrum, which are pro-regenerative. Whereas CD271^+^ activated pericytes may undergo apoptosis between several days to a couple of weeks after activation (Kendall et al., 2009; Siao et al., 2012), M2 macrophages present at later stages of wound healing secrete pro-regenerative factors such as TGF-β, PDGF, VEGF, and EGF (Hesketh et al., 2017), and may thus maintain the wound healing process even after the disappearance of these MSC-like, activated pericytes.

As discussed above, production of the active form of TGF-β appears to be an important way by which MSCs and activated pericytes contribute to the M2 polarization by macrophages, but it is likely that additional molecular events are involved in the crosstalk between these cells *in vivo*. In view of the similarity between activated pericytes and MSCs, mechanisms by which the latter contribute to the acquisition of an M2 phenotype by macrophages *in vitro* may provide useful insights. For example, two chemokines produced by MSCs (and non-cultured pericytes as well), CCL2 and CXCL12, have been shown to form a dimer that contribute to the acquisition of an interleukin 10 (IL-10) producing M2 phenotype by macrophages in experimentally induced colitis in mice, with consequent secretion of IL-10 by bystander T and B cells (Giri et al., 2020). Efferocytosis, the process by which macrophages remove apoptotic cells by phagocytosis, has also been demonstrated to play a role on M2 polarization by MSCs both *in vitro* (Ghahremani Piraghaj et al., 2018) and *in vivo* (Pang et al., 2021). Pang et al. found not only that MSCs trapped in the lungs after systemic infusion undergo apoptosis, but also that apoptotic MSCs infused in mice after ovalbumin-induced asthma induce the acquisition of an immunosuppressive phenotype by alveolar macrophages (Pang et al., 2021). Given that a number of activated pericytes are expected to become apoptotic during the course of wound healing (da Silva Meirelles et al., 2020), it is possible that clearance of these apoptotic cells by macrophages plays a role on M2 polarization of macrophages as well. Finally, MSCs (Kerkelä et al., 2016) and at least one pericyte cell line (Andrade et al., 2008) have been shown to convert extracellular AMP into adenosine, which can lead to the conversion of an M1 to an M2 (M2d) phenotype in macrophages (Ferrante et al., 2013), a mechanism that may be deployed as pericytes become activated during wound healing, particularly if activated T cells are present to convert extracellular ATP released by dead cells into AMP (Kerkelä et al., 2016).

The ability of MSCs to promote M2 polarization in macrophages by priming with interferon-γ (IFN-γ) and tumor necrosis factor (TNF), two cytokines involved in cellular immune responses, has been demonstrated a decade ago (François et al., 2012). It is noteworthy that the ability to contribute to the generation of pro-regenerative macrophages may be further increased in MSCs by exposing them to molecules like LPS prior to extracellular vesicle harvesting and administration (Ti et al., 2015). More recently, the use of apoptotic MSCs to mitigate sepsis by promotion of M2 polarization in macrophages has been demonstrated (Pan et al., 2022), which suggests that even allogeneic MSCs induced to become apoptotic could be used to treat conditions that could be improved by increasing the number of M2 macrophages. Acute conditions that affect the brain, in which neuroinflammation greatly contributes to a negative outcome (da Silva Meirelles et al., 2017; Regner et al., 2017), represent a scenario in which these strategies may be particularly useful. After acute brain injury, local and systemic inflammatory responses initiate early, and play key roles in the secondary injury progression that evolves to neuronal loss and neurological deficits (Kumar et al., 2017). The local inflammatory response following neurotrauma is initiated by microglial cells, which produce EVs loaded with pro-inflammatory molecules that reach and activate additional microglial cells that contribute to a progressively greater neuroinflammatory response (Kumar et al., 2017). Administration of MSCs to rats early after experimental traumatic brain injury has been shown to suppress neuroinflammation and reduce cell death (Zhang et al., 2013). The predominant mode of MSC contribution to brain tissue repair and functional recovery is likely the secretion of EVs such as exosomes containing paracrine molecules (Pan et al., 2017; Yang et al., 2017). In this context, the use of MSC-derived EVs and their miRNA cargo for the treatment of acute brain injuries is also discussed (Xiong et al., 2017). The outlook is that, by using MSCs or MSC-derived EVs to diminish damaging inflammation, a pro-regenerative environment may be established so that endogenous stem and progenitor cells can contribute to repair the affected neural tissue or minimize the damage caused by the injury (Li Y. et al., 2017a; Börger et al., 2017). Evidently, is likely that similar approaches can be used to treat other conditions in which inflammation plays a pivotal role.

## 7 Conclusion

Mesenchymal stromal cells have come a long way since the first accounts of their existence in bone marrow and potential use in regenerative medicine. Through this time, concepts regarding their nature and mechanisms of action in health and disease evolved, as reflected by different names used to call them, and changes in the approaches to harness their therapeutic potential–from building blocks for tissue engineering to factories of biologically active molecules. Today, a relationship between these cells and pericytes is evident, as pericytes have reportedly been shown to give rise to MSCs. While discussing the possible existence of mesenchymal stem cells in the body as discrete populations of pericytes is still valid, doing so does not seem to be more important than advancing the knowledge on the behavior of MSCs derived from pericytes activated during wound healing and on the ability of these cells to secrete trophic and immunomodulatory molecules during acute conditions. The interactions between MSCs and inflammatory cells represent, at this time, an important research front to gain information on the therapeutic properties of these cells. The use of MSC-derived extracellular vesicles will likely become a prevalent approach to treat various conditions, as their delivery *in vivo* is safe and less prone to the complications associated with delivery of cultured MSCs, such as entrapment in capillaries.

## References

[B1] Andrade C. M. B. Roesch G. C. Wink M. R. Guimarães E. L. M. Souza L. F. Jardim F. R. (2008). Activity and expression of ecto-5’-nucleotidase/CD73 are increased during phenotype conversion of a hepatic stellate cell line. Life Sci. 82, 21–29. 10.1016/j.lfs.2007.10.003 18037449

[B2] Awad H. A. Butler D. L. Boivin G. P. Smith F. N. Malaviya P. Huibregtse B. (1999). Autologous mesenchymal stem cell-mediated repair of tendon. Tissue Eng. 5, 267–277. 10.1089/ten.1999.5.267 10434073

[B3] Bartholomew A. Sturgeon C. Siatskas M. Ferrer K. McIntosh K. Patil S. (2002). Mesenchymal stem cells suppress lymphocyte proliferation *in vitro* and prolong skin graft survival *in vivo* . Exp. Hematol. 30, 42–48. 10.1016/s0301-472x(01)00769-x 11823036

[B4] Bianco P. Cossu G. (1999). Uno, nessuno e centomila: Searching for the identity of mesodermal progenitors. Exp. Cell Res. 251, 257–263. 10.1006/excr.1999.4592 10471311

[B5] Bianco P. Riminucci M. Gronthos S. Robey P. G. (2001). Bone marrow stromal stem cells: Nature, biology, and potential applications. Stem Cells 19, 180–192. 10.1634/stemcells.19-3-180 11359943

[B6] Birbrair A. Zhang T. Wang Z.-M. Messi M. L. Enikolopov G. N. Mintz A. (2013). Role of pericytes in skeletal muscle regeneration and fat accumulation. Stem Cells Dev. 22, 2298–2314. 10.1089/scd.2012.0647 23517218 PMC3730538

[B7] Börger V. Bremer M. Ferrer-Tur R. Gockeln L. Stambouli O. Becic A. (2017). Mesenchymal stem/stromal cell-derived extracellular vesicles and their potential as novel immunomodulatory therapeutic agents. Int. J. Mol. Sci. 18, E1450. 10.3390/ijms18071450 PMC5535941 28684664

[B8] Brighton C. T. Hunt R. M. (1997). Early histologic and ultrastructural changes in microvessels of periosteal callus. J. Orthop. Trauma 11, 244–253. 10.1097/00005131-199705000-00002 9258821

[B9] Brighton C. T. Lorich D. G. Kupcha R. Reilly T. M. Jones A. R. Woodbury R. A. (1992). The pericyte as a possible osteoblast progenitor cell. Clin. Orthop. Relat. Res. 275, 287–299. 10.1097/00003086-199202000-00043 1735227

[B10] Bruno S. Grange C. Deregibus M. C. Calogero R. A. Saviozzi S. Collino F. (2009). Mesenchymal stem cell-derived microvesicles protect against acute tubular injury. J. Am. Soc. Nephrol. 20, 1053–1067. 10.1681/ASN.2008070798 19389847 PMC2676194

[B11] Caplan A. I. (2008). All MSCs are pericytes? Cell Stem Cell 3, 229–230. 10.1016/j.stem.2008.08.008 18786406

[B12] Caplan A. I. (1991). Mesenchymal stem cells. J. Orthop. Res. 9, 641–650. 10.1002/jor.1100090504 1870029

[B13] Caplan A. I. (2010). What’s in a name? Tissue Eng. Part A 16, 2415–2417. 10.1089/ten.TEA.2010.0216 20412005

[B14] Cattoretti G. Schiró R. Orazi A. Soligo D. Colombo M. P. (1993). Bone marrow stroma in humans: Anti-nerve growth factor receptor antibodies selectively stain reticular cells *in vivo* and *in vitro* . Blood 81, 1726–1738. 10.1182/blood.v81.7.1726.bloodjournal8171726 7681701

[B15] Churchman S. M. Ponchel F. Boxall S. A. Cuthbert R. Kouroupis D. Roshdy T. (2012). Transcriptional profile of native CD271+ multipotential stromal cells: Evidence for multiple fates, with prominent osteogenic and wnt pathway signaling activity. Arthritis Rheum. 64, 2632–2643. 10.1002/art.34434 22378497

[B16] Crisan M. Yap S. Casteilla L. Chen C.-W. Corselli M. Park T. S. (2008). A perivascular origin for mesenchymal stem cells in multiple human organs. Cell Stem Cell 3, 301–313. 10.1016/j.stem.2008.07.003 18786417

[B17] da Silva Meirelles L. Caplan A. I. Nardi N. B. (2008). In search of the *in vivo* identity of mesenchymal stem cells. Stem Cells 26, 2287–2299. 10.1634/stemcells.2007-1122 18566331

[B18] da Silva Meirelles L. Chagastelles P. C. Nardi N. B. (2006). Mesenchymal stem cells reside in virtually all post-natal organs and tissues. J. Cell Sci. 119, 2204–2213. 10.1242/jcs.02932 16684817

[B19] da Silva Meirelles L. de Deus Wagatsuma V. M. Malta T. M. Bonini Palma P. V. Araújo A. G. Panepucci R. A. (2016). The gene expression profile of non-cultured, highly purified human adipose tissue pericytes: Transcriptomic evidence that pericytes are stem cells in human adipose tissue. Exp. Cell Res. 349, 239–254. 10.1016/j.yexcr.2016.10.017 27789253

[B20] da Silva Meirelles L. Malta T. M. de Deus Wagatsuma V. M. Palma P. V. B. Araújo A. G. Ribeiro Malmegrim K. C. (2015). Cultured human adipose tissue pericytes and mesenchymal stromal cells display a very similar gene expression profile. Stem Cells Dev. 24, 2822–2840. 10.1089/scd.2015.0153 26192741 PMC4653823

[B21] da Silva Meirelles L. Marson R. F. Solari M. I. G. Nardi N. B. (2020). Are liver pericytes just precursors of myofibroblasts in hepatic diseases? Insights from the crosstalk between perivascular and inflammatory cells in liver injury and repair. Cells 9, E188. 10.3390/cells9010188 PMC7017158 31940814

[B22] da Silva Meirelles L. Simon D. Regner A. (2017). Neurotrauma: The crosstalk between neurotrophins and inflammation in the acutely injured brain. Int. J. Mol. Sci. 18, E1082. 10.3390/ijms18051082 PMC5454991 28524074

[B23] Davidoff M. S. Middendorff R. Enikolopov G. Riethmacher D. Holstein A. F. Müller D. (2004). Progenitor cells of the testosterone-producing Leydig cells revealed. J. Cell Biol. 167, 935–944. 10.1083/jcb.200409107 15569711 PMC2172461

[B24] Dellavalle A. Maroli G. Covarello D. Azzoni E. Innocenzi A. Perani L. (2011). Pericytes resident in postnatal skeletal muscle differentiate into muscle fibres and generate satellite cells. Nat. Commun. 2, 499. 10.1038/ncomms1508 21988915

[B25] Dellavalle A. Sampaolesi M. Tonlorenzi R. Tagliafico E. Sacchetti B. Perani L. (2007). Pericytes of human skeletal muscle are myogenic precursors distinct from satellite cells. Nat. Cell Biol. 9, 255–267. 10.1038/ncb1542 17293855

[B26] Dexter T. M. Allen T. D. Lajtha L. G. (1977). Conditions controlling the proliferation of haemopoietic stem cells *in vitro* . J. Cell Physiol. 91, 335–344. 10.1002/jcp.1040910303 301143

[B27] Di Nicola M. Carlo-Stella C. Magni M. Milanesi M. Longoni P. D. Matteucci P. (2002). Human bone marrow stromal cells suppress T-lymphocyte proliferation induced by cellular or nonspecific mitogenic stimuli. Blood 99, 3838–3843. 10.1182/blood.v99.10.3838 11986244

[B28] Dias Moura Prazeres P. H. Sena I. F. G. Borges I. da T. de Azevedo P. O. Andreotti J. P. de Paiva A. E. (2017). Pericytes are heterogeneous in their origin within the same tissue. Dev. Biol. 427, 6–11. 10.1016/j.ydbio.2017.05.001 28479340 PMC6076854

[B29] Diaz-Flores L. Gutierrez R. Gonzalez P. Varela H. (1991). Inducible perivascular cells contribute to the neochondrogenesis in grafted perichondrium. Anat. Rec. 229, 1–8. 10.1002/ar.1092290102 1996774

[B30] Diaz-Flores L. Gutierrez R. Lopez-Alonso A. Gonzalez R. Varela H. (1992). Pericytes as a supplementary source of osteoblasts in periosteal osteogenesis. Clin. Orthop. Relat. Res. 275, 280–286. 10.1097/00003086-199202000-00042 1735226

[B31] Doherty M. J. Ashton B. A. Walsh S. Beresford J. N. Grant M. E. Canfield A. E. (1998). Vascular pericytes express osteogenic potential *in vitro* and *in vivo* . J. Bone Min. Res. 13, 828–838. 10.1359/jbmr.1998.13.5.828 9610747

[B32] Dore-Duffy P. Owen C. Balabanov R. Murphy S. Beaumont T. Rafols J. A. (2000). Pericyte migration from the vascular wall in response to traumatic brain injury. Microvasc. Res. 60, 55–69. 10.1006/mvre.2000.2244 10873515

[B33] Dore-Duffy P. (2008). Pericytes: Pluripotent cells of the blood brain barrier. Curr. Pharm. Des. 14, 1581–1593. 10.2174/138161208784705469 18673199

[B34] Eming S. A. Wynn T. A. Martin P. (2017). Inflammation and metabolism in tissue repair and regeneration. Science 356, 1026–1030. 10.1126/science.aam7928 28596335

[B35] Farrington-Rock C. Crofts N. J. Doherty M. J. Ashton B. A. Griffin-Jones C. Canfield A. E. (2004). Chondrogenic and adipogenic potential of microvascular pericytes. Circulation 110, 2226–2232. 10.1161/01.CIR.0000144457.55518.E5 15466630

[B36] Feng J. Mantesso A. De Bari C. Nishiyama A. Sharpe P. T. (2011). Dual origin of mesenchymal stem cells contributing to organ growth and repair. Proc. Natl. Acad. Sci. U. S. A. 108, 6503–6508. 10.1073/pnas.1015449108 21464310 PMC3081015

[B37] Ferrante C. J. Leibovich S. J. (2012). Regulation of macrophage polarization and wound healing. Adv. Wound Care (New Rochelle) 1, 10–16. 10.1089/wound.2011.0307 24527272 PMC3623587

[B38] Ferrante C. J. Pinhal-Enfield G. Elson G. Cronstein B. N. Hasko G. Outram S. (2013). The adenosine-dependent angiogenic switch of macrophages to an M2-like phenotype is independent of interleukin-4 receptor alpha (IL-4Rα) signaling. Inflammation 36, 921–931. 10.1007/s10753-013-9621-3 23504259 PMC3710311

[B39] Flores-Figueroa E. Varma S. Montgomery K. Greenberg P. L. Gratzinger D. (2012). Distinctive contact between CD34+ hematopoietic progenitors and CXCL12+ CD271+ mesenchymal stromal cells in benign and myelodysplastic bone marrow. Lab. Invest. 92, 1330–1341. 10.1038/labinvest.2012.93 22710983

[B40] FrançoisM.Romieu-MourezR.LiM.GalipeauJ. (2012). Human MSC suppression correlates with cytokine induction of indoleamine 2,3-dioxygenase and bystander M2 macrophage differentiation. Mol. Ther. 20, 187–195. 10.1038/mt.2011.189 21934657

[B41] Friedenstein A. J. Chailakhjan R. K. Lalykina K. S. (1970). The development of fibroblast colonies in monolayer cultures of Guinea-pig bone marrow and spleen cells. Cell Tissue Kinet. 3, 393–403. 10.1111/j.1365-2184.1970.tb00347.x 5523063

[B42] Friedenstein A. J. Chailakhyan R. K. Gerasimov U. V. (1987). Bone marrow osteogenic stem cells: *In vitro* cultivation and transplantation in diffusion chambers. Cell Tissue Kinet. 20, 263–272. 10.1111/j.1365-2184.1987.tb01309.x 3690622

[B43] Friedenstein A. J. Chailakhyan R. K. Latsinik N. V. Panasyuk A. F. Keiliss-Borok I. V. (1974a). Stromal cells responsible for transferring the microenvironment of the hemopoietic tissues. Cloning *in vitro* and retransplantation *in vivo* . Transplantation 17, 331–340. 10.1097/00007890-197404000-00001 4150881

[B44] Friedenstein A. J. Deriglasova U. F. Kulagina N. N. Panasuk A. F. Rudakowa S. F. Luriá E. A. (1974b). Precursors for fibroblasts in different populations of hematopoietic cells as detected by the *in vitro* colony assay method. Exp. Hematol. 2, 83–92. 4455512

[B45] Fukuda K. (2001). Development of regenerative cardiomyocytes from mesenchymal stem cells for cardiovascular tissue engineering. Artif. Organs 25, 187–193. 10.1046/j.1525-1594.2001.025003187.x 11284885

[B46] Ganguly P. El-Jawhari J. J. Burska A. N. Ponchel F. Giannoudis P. V. Jones E. A. (2019). The analysis of *in vivo* aging in human bone marrow mesenchymal stromal cells using colony-forming unit-fibroblast assay and the CD45lowCD271+ phenotype. Stem Cells Int. 2019, 5197983. 10.1155/2019/5197983 31467563 PMC6701348

[B47] Gerhardt H. Betsholtz C. (2003). Endothelial-pericyte interactions in angiogenesis. Cell Tissue Res. 314, 15–23. 10.1007/s00441-003-0745-x 12883993

[B48] Ghahremani Piraghaj M. Soudi S. Ghanbarian H. Bolandi Z. Namaki S. Hashemi S. M. (2018). Effect of efferocytosis of apoptotic mesenchymal stem cells (MSCs) on C57BL/6 peritoneal macrophages function. Life Sci. 212, 203–212. 10.1016/j.lfs.2018.09.052 30287233

[B49] Giri J. Das R. Nylen E. Chinnadurai R. Galipeau J. (2020). CCL2 and CXCL12 derived from mesenchymal stromal cells cooperatively polarize IL-10+ tissue macrophages to mitigate gut injury. Cell Rep. 30, 1923–1934.e4. 10.1016/j.celrep.2020.01.047 32049021 PMC7043065

[B50] Gnecchi M. He H. Liang O. D. Melo L. G. Morello F. Mu H. (2005). Paracrine action accounts for marked protection of ischemic heart by Akt-modified mesenchymal stem cells. Nat. Med. 11, 367–368. 10.1038/nm0405-367 15812508

[B51] Goshima J. Goldberg V. M. Caplan A. I. (1991). The osteogenic potential of culture-expanded rat marrow mesenchymal cells assayed *in vivo* in calcium phosphate ceramic blocks. Clin. Orthop. Relat. Res. 262, 298–311. 10.1097/00003086-199101000-00038 1984928

[B52] Grande D. A. Southerland S. S. Manji R. Pate D. W. Schwartz R. E. Lucas P. A. (1995). Repair of articular cartilage defects using mesenchymal stem cells. Tissue Eng. 1, 345–353. 10.1089/ten.1995.1.345 19877898

[B53] Grant R. I. Hartmann D. A. Underly R. G. Berthiaume A.-A. Bhat N. R. Shih A. Y. (2019). Organizational hierarchy and structural diversity of microvascular pericytes in adult mouse cortex. J. Cereb. Blood Flow. Metab. 39, 411–425. 10.1177/0271678X17732229 28933255 PMC6399730

[B54] Guillén M. I. Platas J. Pérez Del Caz M. D. Mirabet V. Alcaraz M. J. (2018). Paracrine anti-inflammatory effects of adipose tissue-derived mesenchymal stem cells in human monocytes. Front. Physiol. 9, 661. 10.3389/fphys.2018.00661 29904354 PMC5990614

[B55] Guimarães-Camboa N. Cattaneo P. Sun Y. Moore-Morris T. Gu Y. Dalton N. D. (2017). Pericytes of multiple organs do not behave as mesenchymal stem cells *in vivo* . Cell Stem Cell 20, 345–359.e5. 10.1016/j.stem.2016.12.006 28111199 PMC5337131

[B56] Hejbøl E. K. Hajjaj M. A. Nielsen O. Schrøder H. D. (2019). Marker expression of interstitial cells in human skeletal muscle: An immunohistochemical study. J. Histochem Cytochem 67, 825–844. 10.1369/0022155419871033 31411936 PMC6824003

[B57] Hesketh M. Sahin K. B. West Z. E. Murray R. Z. (2017). Macrophage phenotypes regulate scar formation and chronic wound healing. Int. J. Mol. Sci. 18, E1545. 10.3390/ijms18071545 PMC5536033 28714933

[B58] Horwitz E. M. Le Blanc K. Dominici M. Mueller I. Slaper-Cortenbach I. Marini F. C. (2005). Clarification of the nomenclature for MSC: The international society for cellular Therapy position statement. Cytotherapy 7, 393–395. 10.1080/14653240500319234 16236628

[B59] Humphreys B. D. Lin S.-L. Kobayashi A. Hudson T. E. Nowlin B. T. Bonventre J. V. (2010). Fate tracing reveals the pericyte and not epithelial origin of myofibroblasts in kidney fibrosis. Am. J. Pathol. 176, 85–97. 10.2353/ajpath.2010.090517 20008127 PMC2797872

[B60] Hung C. F. Mittelsteadt K. L. Brauer R. McKinney B. L. Hallstrand T. S. Parks W. C. (2017). Lung pericyte-like cells are functional interstitial immune sentinel cells. Am. J. Physiol. Lung Cell Mol. Physiol. 312, L556–L567. 10.1152/ajplung.00349.2016 28188224 PMC5407093

[B61] Inoue O. Usui S. Takashima S.-I. Nomura A. Yamaguchi K. Takeda Y. (2019). Diabetes impairs the angiogenic capacity of human adipose-derived stem cells by reducing the CD271+ subpopulation in adipose tissue. Biochem. Biophys. Res. Commun. 517, 369–375. 10.1016/j.bbrc.2019.07.081 31362891

[B62] Julien A. Kanagalingam A. Martínez-Sarrà E. Megret J. Luka M. Ménager M. (2021). Direct contribution of skeletal muscle mesenchymal progenitors to bone repair. Nat. Commun. 12, 2860. 10.1038/s41467-021-22842-5 34001878 PMC8128920

[B63] Kaukua N. Shahidi M. K. Konstantinidou C. Dyachuk V. Kaucka M. Furlan A. (2014). Glial origin of mesenchymal stem cells in a tooth model system. Nature 513, 551–554. 10.1038/nature13536 25079316

[B64] Kendall T. J. Hennedige S. Aucott R. L. Hartland S. N. Vernon M. A. Benyon R. C. (2009). p75 Neurotrophin receptor signaling regulates hepatic myofibroblast proliferation and apoptosis in recovery from rodent liver fibrosis. Hepatology 49, 901–910. 10.1002/hep.22701 19072833

[B65] Kerkelä E. Laitinen A. Räbinä J. Valkonen S. Takatalo M. Larjo A. (2016). Adenosinergic immunosuppression by human mesenchymal stromal cells requires Co-operation with T cells. Stem Cells 34, 781–790. 10.1002/stem.2280 26731338

[B66] Kinnaird T. Stabile E. Burnett M. S. Lee C. W. Barr S. Fuchs S. (2004a). Marrow-derived stromal cells express genes encoding a broad spectrum of arteriogenic cytokines and promote *in vitro* and *in vivo* arteriogenesis through paracrine mechanisms. Circ. Res. 94, 678–685. 10.1161/01.RES.0000118601.37875.AC 14739163

[B67] Kinnaird T. Stabile E. Burnett M. S. Shou M. Lee C. W. Barr S. (2004b). Local delivery of marrow-derived stromal cells augments collateral perfusion through paracrine mechanisms. Circulation 109, 1543–1549. 10.1161/01.CIR.0000124062.31102.57 15023891

[B68] Kramann R. Schneider R. K. DiRocco D. P. Machado F. Fleig S. Bondzie P. A. (2015). Perivascular Gli1+ progenitors are key contributors to injury-induced organ fibrosis. Cell Stem Cell 16, 51–66. 10.1016/j.stem.2014.11.004 25465115 PMC4289444

[B69] Krampera M. Glennie S. Dyson J. Scott D. Laylor R. Simpson E. (2003). Bone marrow mesenchymal stem cells inhibit the response of naive and memory antigen-specific T cells to their cognate peptide. Blood 101, 3722–3729. 10.1182/blood-2002-07-2104 12506037

[B70] Kumar A. Stoica B. A. Loane D. J. Yang M. Abulwerdi G. Khan N. (2017). Microglial-derived microparticles mediate neuroinflammation after traumatic brain injury. J. Neuroinflammation 14, 47. 10.1186/s12974-017-0819-4 28292310 PMC5351060

[B71] Lanotte M. Allen T. D. Dexter T. M. (1981). Histochemical and ultrastructural characteristics of a cell line from human bone-marrow stroma. J. Cell Sci. 50, 281–297. 10.1242/jcs.50.1.281 7320070

[B72] Le Blanc K. Rasmusson I. Sundberg B. Götherström C. Hassan M. Uzunel M. (2004). Treatment of severe acute graft-versus-host disease with third party haploidentical mesenchymal stem cells. Lancet 363, 1439–1441. 10.1016/S0140-6736(04)16104-7 15121408

[B73] Li Y.-W. Zhang C. Sheng Q.-J. Bai H. Ding Y. Dou X.-G. (2017b). Mesenchymal stem cells rescue acute hepatic failure by polarizing M2 macrophages. World J. Gastroenterol. 23, 7978–7988. 10.3748/wjg.v23.i45.7978 29259373 PMC5725292

[B74] Li Y. Yang Y.-Y. Ren J.-L. Xu F. Chen F.-M. Li A. (2017a). Exosomes secreted by stem cells from human exfoliated deciduous teeth contribute to functional recovery after traumatic brain injury by shifting microglia M1/M2 polarization in rats. Stem Cell Res. Ther. 8, 198. 10.1186/s13287-017-0648-5 28962585 PMC5622448

[B75] Lo Sicco C. Reverberi D. Balbi C. Ulivi V. Principi E. Pascucci L. (2017). Mesenchymal stem cell-derived extracellular vesicles as mediators of anti-inflammatory effects: Endorsement of macrophage polarization. Stem Cells Transl. Med. 6, 1018–1028. 10.1002/sctm.16-0363 28186708 PMC5442783

[B76] Maes C. Kobayashi T. Selig M. K. Torrekens S. Roth S. I. Mackem S. (2010). Osteoblast precursors, but not mature osteoblasts, move into developing and fractured bones along with invading blood vessels. Dev. Cell 19, 329–344. 10.1016/j.devcel.2010.07.010 20708594 PMC3540406

[B77] Mantovani A. Sozzani S. Locati M. Allavena P. Sica A. (2002). Macrophage polarization: Tumor-associated macrophages as a paradigm for polarized M2 mononuclear phagocytes. Trends Immunol. 23, 549–555. 10.1016/s1471-4906(02)02302-5 12401408

[B78] Martinez F. O. Sica A. Mantovani A. Locati M. (2008). Macrophage activation and polarization. Front. Biosci. 13, 453–461. 10.2741/2692 17981560

[B79] Mastrolia I. Foppiani E. M. Murgia A. Candini O. Samarelli A. V. Grisendi G. (2019). Challenges in clinical development of mesenchymal stromal/stem cells: Concise review. Stem Cells Transl. Med. 8, 1135–1148. 10.1002/sctm.19-0044 31313507 PMC6811694

[B80] Meisel R. Zibert A. Laryea M. Göbel U. Däubener W. Dilloo D. (2004). Human bone marrow stromal cells inhibit allogeneic T-cell responses by indoleamine 2,3-dioxygenase-mediated tryptophan degradation. Blood 103, 4619–4621. 10.1182/blood-2003-11-3909 15001472

[B81] Méndez-Ferrer S. Michurina T. V. Ferraro F. Mazloom A. R. Macarthur B. D. Lira S. A. (2010). Mesenchymal and haematopoietic stem cells form a unique bone marrow niche. Nature 466, 829–834. 10.1038/nature09262 20703299 PMC3146551

[B82] Minutti C. M. Modak R. V. Macdonald F. Li F. Smyth D. J. Dorward D. A. (2019). A macrophage-pericyte Axis directs tissue restoration via amphiregulin-induced transforming growth factor beta activation. Immunity 50, 645–654.e6. 10.1016/j.immuni.2019.01.008 30770250 PMC6436929

[B83] Navarro R. Compte M. Álvarez-Vallina L. Sanz L. (2016). Immune regulation by pericytes: Modulating innate and adaptive immunity. Front. Immunol. 7, 480. 10.3389/fimmu.2016.00480 27867386 PMC5095456

[B84] Owen M. E. Cavé J. Joyner C. J. (1987). Clonal analysis *in vitro* of osteogenic differentiation of marrow CFU-F. J. Cell Sci. 87, 731–738. 10.1242/jcs.87.5.731 3499442

[B85] Owen M. (1988). Marrow stromal stem cells. J. Cell Sci. Suppl. 10, 63–76. 10.1242/jcs.1988.supplement_10.5 3077943

[B86] Pan Y.-B. Sun Z.-L. Feng D.-F. (2017). The role of MicroRNA in traumatic brain injury. Neuroscience 367, 189–199. 10.1016/j.neuroscience.2017.10.046 29113926

[B87] Pan Y. Li J. Wang J. Jiang Q. Yang J. Dou H. (2022). Ferroptotic MSCs protect mice against sepsis via promoting macrophage efferocytosis. Cell Death Dis. 13, 825. 10.1038/s41419-022-05264-z 36163182 PMC9512818

[B88] Pang S. H. M. D’Rozario J. Mendonca S. Bhuvan T. Payne N. L. Zheng D. (2021). Mesenchymal stromal cell apoptosis is required for their therapeutic function. Nat. Commun. 12, 6495. 10.1038/s41467-021-26834-3 34764248 PMC8586224

[B89] Pierantozzi E. Badin M. Vezzani B. Curina C. Randazzo D. Petraglia F. (2015). Human pericytes isolated from adipose tissue have better differentiation abilities than their mesenchymal stem cell counterparts. Cell Tissue Res. 361, 769–778. 10.1007/s00441-015-2166-z 25820673

[B90] Pittenger M. F. Mackay A. M. Beck S. C. Jaiswal R. K. Douglas R. Mosca J. D. (1999). Multilineage potential of adult human mesenchymal stem cells. Science 284, 143–147. 10.1126/science.284.5411.143 10102814

[B91] Proebstl D. Voisin M.-B. Woodfin A. Whiteford J. D’Acquisto F. Jones G. E. (2012). Pericytes support neutrophil subendothelial cell crawling and breaching of venular walls *in vivo* . J. Exp. Med. 209, 1219–1234. 10.1084/jem.20111622 22615129 PMC3371725

[B92] Regner A. da Silva Meirelles L. Simon D. (2017). Traumatic penumbra: Opportunities for neuroprotective and neurorestorative processes *traumatic brain injury - pathobiology, advanced Diagnostics and acute management* (london: IntechOpen). Available at: https://www.intechopen.com/chapters/58061.

[B93] Richardson R. L. Hausman G. J. Campion D. R. (1982). Response of pericytes to thermal lesion in the inguinal fat pad of 10-day-old rats. Acta Anat. (Basel) 114, 41–57. 10.1159/000145577 7148377

[B94] Sacchetti B. Funari A. Michienzi S. Di Cesare S. Piersanti S. Saggio I. (2007). Self-renewing osteoprogenitors in bone marrow sinusoids can organize a hematopoietic microenvironment. Cell 131, 324–336. 10.1016/j.cell.2007.08.025 17956733

[B95] Sacchetti B. Funari A. Remoli C. Giannicola G. Kogler G. Liedtke S. (2016). No identical “mesenchymal stem cells” at different times and sites: Human committed progenitors of distinct origin and differentiation potential are incorporated as adventitial cells in microvessels. Stem Cell Rep. 6, 897–913. 10.1016/j.stemcr.2016.05.011 PMC4912436 27304917

[B96] Saito T. Dennis J. E. Lennon D. P. Young R. G. Caplan A. I. (1995). Myogenic expression of mesenchymal stem cells within myotubes of mdx mice *in vitro* and *in vivo* . Tissue Eng. 1, 327–343. 10.1089/ten.1995.1.327 19877897

[B97] Siao C.-J. Lorentz C. U. Kermani P. Marinic T. Carter J. McGrath K. (2012). ProNGF, a cytokine induced after myocardial infarction in humans, targets pericytes to promote microvascular damage and activation. J. Exp. Med. 209, 2291–2305. 10.1084/jem.20111749 23091165 PMC3501352

[B98] Stark K. Eckart A. Haidari S. Tirniceriu A. Lorenz M. von Brühl M.-L. (2013). Capillary and arteriolar pericytes attract innate leukocytes exiting through venules and “instruct” them with pattern-recognition and motility programs. Nat. Immunol. 14, 41–51. 10.1038/ni.2477 23179077

[B99] Supakul S. Yao K. Ochi H. Shimada T. Hashimoto K. Sunamura S. (2019). Pericytes as a source of osteogenic cells in bone fracture healing. Int. J. Mol. Sci. 20, E1079. 10.3390/ijms20051079 PMC6429337 30832329

[B100] Tang W. Zeve D. Suh J. M. Bosnakovski D. Kyba M. Hammer R. E. (2008). White fat progenitor cells reside in the adipose vasculature. Science 322, 583–586. 10.1126/science.1156232 18801968 PMC2597101

[B101] Tang Y. L. Zhao Q. Qin X. Shen L. Cheng L. Ge J. (2005). Paracrine action enhances the effects of autologous mesenchymal stem cell transplantation on vascular regeneration in rat model of myocardial infarction. Ann. Thorac. Surg. 80, 229. 10.1016/j.athoracsur.2005.02.072 15975372

[B102] Tavassoli M. Crosby W. H. (1968). Transplantation of marrow to extramedullary sites. Science 161, 54–56. 10.1126/science.161.3836.54 4871792

[B103] Ti D. Hao H. Tong C. Liu J. Dong L. Zheng J. (2015). LPS-preconditioned mesenchymal stromal cells modify macrophage polarization for resolution of chronic inflammation via exosome-shuttled let-7b. J. Transl. Med. 13, 308. 10.1186/s12967-015-0642-6 26386558 PMC4575470

[B104] Till J. E. McCulloch E. A. (1961). A direct measurement of the radiation sensitivity of normal mouse bone marrow cells. Radiat. Res. 14, 213–AV7. 10.1667/rrav01.1 13776896

[B105] Viswanathan S. Shi Y. Galipeau J. Krampera M. Leblanc K. Martin I. (2019). Mesenchymal stem versus stromal cells: International society for cell and gene Therapy (ISCT®) mesenchymal stromal cell committee position statement on nomenclature. Cytotherapy 21, 1019–1024. 10.1016/j.jcyt.2019.08.002 31526643

[B106] Xiong Y. Mahmood A. Chopp M. (2017). Emerging potential of exosomes for treatment of traumatic brain injury. Neural Regen. Res. 12, 19–22. 10.4103/1673-5374.198966 28250732 PMC5319225

[B107] Yang Y. Ye Y. Su X. He J. Bai W. He X. (2017). MSCs-derived exosomes and neuroinflammation, neurogenesis and Therapy of traumatic brain injury. Front. Cell Neurosci. 11, 55. 10.3389/fncel.2017.00055 28293177 PMC5329010

[B108] Yianni V. Sharpe P. T. (2018). Molecular programming of perivascular stem cell precursors. Stem Cells 36, 1890–1904. 10.1002/stem.2895 30068019

[B109] Yin Y. Hao H. Cheng Y. Zang L. Liu J. Gao J. (2018). Human umbilical cord-derived mesenchymal stem cells direct macrophage polarization to alleviate pancreatic islets dysfunction in type 2 diabetic mice. Cell Death Dis. 9, 760. 10.1038/s41419-018-0801-9 29988034 PMC6037817

[B110] Zhang R. Liu Y. Yan K. Chen L. Chen X.-R. Li P. (2013). Anti-inflammatory and immunomodulatory mechanisms of mesenchymal stem cell transplantation in experimental traumatic brain injury. J. Neuroinflammation 10, 106. 10.1186/1742-2094-10-106 23971414 PMC3765323

[B111] Zhou B. O. Yue R. Murphy M. M. Peyer J. G. Morrison S. J. (2014). Leptin-receptor-expressing mesenchymal stromal cells represent the main source of bone formed by adult bone marrow. Cell Stem Cell 15, 154–168. 10.1016/j.stem.2014.06.008 24953181 PMC4127103

